# Experimental estimation of the longitudinal component of a highly focused electromagnetic field

**DOI:** 10.1038/s41598-021-97164-z

**Published:** 2021-09-09

**Authors:** David Maluenda, Marcos Aviñoá, Kavan Ahmadi, Rosario Martínez-Herrero, Artur Carnicer

**Affiliations:** 1 Departament de Física Aplicada, Universitat de Barcelona (UB), Martí i Franquès 1, 08028 Barcelona, Spain; 2 Departamento de Óptica, Facultad de Ciencias Físicas, Universidad Complutense de Madrid, Ciudad Universitaria, 28040 Madrid, Spain

**Keywords:** Optical physics, Micro-optics

## Abstract

The detection of the longitudinal component of a highly focused electromagnetic beam is not a simple task. Although in recent years several methods have been reported in the literature, this measure is still not routinely performed. This paper describes a method that allows us to estimate and visualize the longitudinal component of the field in a relatively simple way. First, we measure the transverse components of the focused field in several planes normal to the optical axis. Then, we determine the complex amplitude of the two transverse field components: the phase is obtained using a phase recovery algorithm, while the phase difference between the two components is determined from the Stokes parameters. Finally, the longitudinal component is estimated using the Gauss’s theorem. Experimental results show an excellent agreement with theoretical predictions.

## Introduction

Highly focused beams with a non-homogeneous distribution of polarization have been studied over the last years^1–12^ because of its potential applications in many fields such as microscopy, nonlinear optics and plasmonics^13–21^. The input vector field^22,23^ at the entrance pupil of the focusing system should be tailored according to the specific requirements of the problem. Very often, a combination of diffractive, interferometric and holographic techniques is used in order to achieve full control of the complex amplitude and polarization distributions of the input field. See, for instance^24–34^.

Arguably, the most intriguing property of focused beams is the longitudinal component that, under certain conditions, can gather more energy than the transverse one. As it is well-known, the longitudinal component cannot be recorded using a conventional imaging system^35,36^. So far, its direct observation remains a challenging task. Several techniques for direct measuring of the longitudinal component have been described, but they typically are complex or specific for the field of application where they have been designed^37–39^. One of the most promising methods are those based on scanning near-field optical microscopy (SNOM)^40–42^. Nevertheless, the performance and the energy ratio between transverse and longitudinal components collected by SNOM significantly depends on the type and quality of SNOM probes^43–46^. Even though, since the transverse components are easily accessible, an alternative can be found by estimating the longitudinal component using the Gauss’ theorem, provided the complex amplitude and polarization of the transverse field are known^36,47^. Indeed, the amplitude can be inferred from a conventional camera, while the phase cannot be accessed in a straightforward way. Phase distribution can be inferred using interferometric techniques^48,49^, but the use of an interferometric setup increases the complexity of the optical system. In contrast, the well-known Gerchberg–Saxton algorithm provides a way to retrieve the phase distribution through an iterative Fourier Transform algorithm with imposed constraints. In the case of free space propagation, these constraints naturally translate into two plane irradiance distributions. Later on, more general and robust methods were proposed, such as the hybrid input–output algorithm^50–52^ or other derivative approaches that can be used when the planes are computationally connected by means of the Fresnel transfer function^53^. Furthermore, the relative phase between the two transverse components can be recovered by means of polarimetric analysis based on the measurement of Stokes images in the observation planes^54^. Once the electric field is determined at a given plane, the beam can be propagated to any new location^55^. Recently, some authors are considering applications on shaping the longitudinal component for beam design^56^ or in information optics^21^. In the present paper we propose a method to estimate the longitudinal components in the focal region of a highly focused beam based on the information contained on complex amplitudes of the corresponding transverse part.

This paper is organized as follows: first, we describe the theoretical background for the longitudinal component estimation. Thereafter, in "Methods", we describe the adapted algorithm used to retrieve the phase of an electromagnetic field, and explain the experimental setup. The estimation of the longitudinal component can be found in "Results and discussion" and finally, our conclusions are summarized in the "Concluding remarks" section.

## Longitudinal and transverse components of an electromagnetic field

The electromagnetic field in free space must satisfy Maxwell’s Equations, specifically, the Gauss’ theorem

1 $$\begin{aligned} \mathbf {\nabla }\cdot \mathbf {E}(\mathbf {r}) = 0 , \end{aligned}$$

where $\mathbf {E}(\mathbf {{\mathbf {r}}})$ is the electric field and $\mathbf {r}$ is the position vector. Time dependence is dropped since in this work we only consider quasi-monochromatic waves. For plane waves, the Gauss’ theorem represents the transverse condition for the electromagnetic field: the polarization direction of the beam is perpendicular to the direction of propagation. However, non-homogeneous fields can be understood as being composed by a set of plane waves traveling in different directions. Therefore, the direction of propagation is not perfectly defined, and we cannot strictly talk about transverse waves.

Without loss of generality, we consider the propagation of electromagnetic waves with respect to a reference axis, say the *z* axis. We split the electromagnetic field $\mathbf {E}(\mathbf {r})$ in the following way

2 $$\begin{aligned} \mathbf {E}(\mathbf {r}) = \mathbf {E}_\perp (\mathbf {r}) + E_z(\mathbf {r})\mathbf {e}_z, \end{aligned}$$

where $\mathbf {E}_\perp (\mathbf {r})$ and $E_z(\mathbf {r})$ are the transverse and parallel components to the *z* axis respectively, and $\mathbf {e}_z$ is the unit vector in the direction of the *z* axis. Introducing Eq. (2) into Eq. (1) we obtain the following identity

3 $$\begin{aligned} \mathbf {\nabla }_\perp \cdot \mathbf {E}_\perp (\mathbf {r}) + \frac{\partial E_z(\mathbf {r})}{\partial z} = 0 , \end{aligned}$$

with $\mathbf {\nabla }_\perp = \mathbf {e}_x\frac{\partial }{\partial x} + \mathbf {e}_y\frac{\partial }{\partial y}$ and $\mathbf {e}_x, \mathbf {e}_y, \mathbf {e}_z$ is a orthogonal triad of unit vectors. If we consider each component of the electromagnetic field as composed by a superposition of plane waves^57^,where $\mathbf {k}_\perp =(k_x, k_y)$ and $k_z$ are the transverse and longitudinal wave-vectors, respectively, satisfying $k^2 = k_\perp ^2+k_z^2$:

5 $$\begin{aligned} k_z= & {} \sqrt{k^2-k_\perp ^2} \quad \mathrm {if} \quad k_\perp ^2 \le k^2 \end{aligned}$$

6 $$\begin{aligned} k_z= & {} i \sqrt{k_\perp ^2 - k^2} \quad \mathrm {if} \quad k_\perp ^2 > k^2 . \end{aligned}$$

In the present paper, we only consider the case $k_z$ real due to the experiments we are carrying out. $\hat{\mathbf {E}}_\perp (\mathbf {k}_\perp ; z), {\hat{E}}_z(\mathbf {k}_\perp ;z)$ are the plane wave spectra of the transverse and longitudinal components, respectively. Introducing Eqs. (4)(a–b) in Eq. (3), we obtain the equality

7 $$\begin{aligned} i\mathbf {k}_\perp \cdot \hat{\mathbf {E}}_\perp (\mathbf {k}_\perp ; z) + \frac{\partial {\hat{E}}_z(\mathbf {k}_\perp ; z)}{\partial z} = 0. \end{aligned}$$

Now, as we are considering waves propagating through free space, each Cartesian component satisfies its own Helmholtz equation,

8 $$\begin{aligned} \nabla ^2 E_i(\mathbf {r}) + k^2E_i(\mathbf {r}) = 0, \end{aligned}$$

where $i=x, y, z$. Using the decomposition into plane waves of Eq. (4) in the Helmholtz equation (Eq. (8)), we obtain

9 $$\begin{aligned} \frac{\partial ^2 {\hat{E}}_i(\mathbf {k}_\perp ; z)}{\partial z^2} + k_z^2 {\hat{E}}_i(\mathbf {k}_\perp ; z) = 0 \end{aligned}$$

with general solution

10 $$\begin{aligned} \hat{E_i}(\mathbf {k}_\perp ; z) = \hat{E_i}(\mathbf {k}_\perp ; z=0)\mathrm {e}^{ik_z z}. \end{aligned}$$

Introducing Eq. (10) into Eq. (7) we obtain at once

11 $$\begin{aligned} {\hat{E}}_z(\mathbf {k}_\perp ; z=0) = -\frac{\mathbf {k}_\perp \cdot \hat{\mathbf {E}}_\perp (\mathbf {k}_\perp ; z=0)}{k_z}. \end{aligned}$$

Finally, the longitudinal component in real space is just the inverse Fourier Transform of this spectrum, multiplied by the complex factor $\mathrm {e}^{ik_z z}$

12 $$\begin{aligned} E_z(\mathbf {r}) = -\frac{1}{4\pi ^2}\mathop \int \limits _{k_\perp ^2 \le k^2} \frac{\mathbf {k}_\perp \cdot \hat{\mathbf {E}}_\perp (\mathbf {k}_\perp ; z=0)}{k_z} \mathrm {e}^{ik_z z} \mathrm {e}^{i\mathbf {k}_\perp \cdot \mathbf {r}}d^2k_\perp . \end{aligned}$$

Therefore, the longitudinal component of the electromagnetic field can be written in terms of just the transverse component, up to an unimodular complex factor. The shape of the longitudinal component is greatly dependent on the polarization of the transverse component, through the dot product. For example, two fields with the same complex amplitude, but different polarizations result in two different z components. Equation (12) is the key concept of this work.

## Methods

### Phase recovery algorithm

To recover the longitudinal component of an electromagnetic field by means of Eq. (12), it is necessary to determine the complex amplitudes of the transverse components. However, only the irradiance of the field can be recorded without the use of specific techniques such as holography. However, thanks to the mathematical properties of the electromagnetic fields, a variety of methods to recover the phase by means of irradiance measurements have been developed^58,59^. Among them, iterative algorithms such as the Hybrid Input–Output^50^, might be suitable for the estimation of the longitudinal component.

In order to obtain a fair estimation of the phase of the beam, we record the modulus of the electromagnetic field at *P* different planes perpendicular to the $z-$axis $z_j$ $(j=1,\ldots ,P$),

13 $$\begin{aligned} A_j^2 = |E_i(x, y, z_j) |^2. \end{aligned}$$

with $i=x,y$. Note we should determine the phase associated to each polarization component independently and thus, the procedure should be repeated twice. Complex amplitudes $U_j = A_j\mathrm {e}^{i\phi _j}$ are obtained by means of the following procedure:

1. Assign an initial phase estimation for the first modulus, $U_1 = A_1 \mathrm {e}^{i\phi _1}$.
2. Propagate complex amplitude $U_1$ to plane $z_2$ using a Fresnel propagation. The modulus is discarded, and the phase is assigned to the experimental measure of the irradiance.
3. Repeat the previous step until we reach the final plane, *P*
4. Back-propagate $U_P$ to the first plane. This gives us the next estimation for the initial phase $\mathrm {e}^{i\phi _1}$. The error between the recovered modulus and the experimental one is measured at this step. This process is repeated until the error measure arrives at the prescribed value and/or the measure stagnates.

This kind of iterative algorithms have two main drawbacks: slow rate of convergence and stagnation at local minima of the error function^59,60^. We address each of these problems separately in the following subsections.

#### Propagation method and local minima evasion

The propagation of the electromagnetic field is performed using the angular spectrum of plane waves and the free space transfer function (see Eqs. (4) and (10))^55,57^. Note that other propagation methods could be used as long as the size of the window is not modified^61,62^. The relationship between two planes separated a distance *z* is

14 $$\begin{aligned} U(x, y, z) = \frac{1}{4\pi ^2}\mathop {\iint }\limits _{k_\perp ^2 \le k^2} {\hat{U}}(k_x, k_y; 0)\mathrm {e}^{ik_z z} \mathrm {e}^{i(k_x x+k_y y)} dk_x dk_y \end{aligned}$$

where ${\hat{U}}(k_x, k_y; 0)$ is the spectrum at the first plane.

Recorded moduli might contain a certain amount of noise. This means that the calculated spectra from the experimental recordings have non-zero high frequency components. Moreover, most of the recorded amplitudes have values near to the lower end of the dynamic range of the sensor. These two effects combined can produce the iterative algorithm to prioritize the fitting of noise instead of the actual beam values.

To avoid this effect, we limit the extent of the field spectra. The limiting frequency is estimated by considering the Fourier transforms of the recorded irradiance $A_j^2$ distributions. Because the maximum frequency extent of the irradiance is twice the cutoff frequency of the complex amplitude, in our calculation we set the radius of the support region by considering the frequency for which the Fourier Transform of the irradiance falls to a value close to zero.

#### Acceleration of the convergence speed

Iterative algorithms, although robust, display slow rates of convergence. To overcome this limitation, we use the ad hoc acceleration procedure developed by Biggs and Andrews^63^. This algorithm is independent of the exact shape of the phase recovery method and can be used with any iterative method.

The acceleration algorithm is implemented as follows. We define the following parameterswhere $x_k$ is the estimation for the initial phase at the *k*th iteration, $h_k$ is the difference between the current phase and the previous one, $y_k$ is the estimation for the next value of the phase at the *k*th iteration, and $\alpha _k$ is the *k*th iteration acceleration parameter, explained below.

The next value of the phase $x_{k+1}$ is determined using $g_k$, a parameter defined as the difference between $\psi (y_k)$ and $y_k$; $\psi (\cdot )$ represents a single iteration of the phase recovering algorithm. Note we work with complex exponentials to ensure a robust convergence: the phase has a branching point at $2\pi$ which would create meaningless gradients or oscillations around this point.

The definition of the acceleration parameter is^63^

16 $$\begin{aligned} \alpha _k = \mathfrak {R}\left[ {\frac{\sum g_{k-1}^* g_{k-2}}{\sum g_{k-2}^* g_{k-2}},}\right] \end{aligned}$$

where the summation is taken over all values of the elements of $g_k$ and $^*$ denotes complex conjugate. We have introduced the symbol $\mathfrak {R}$, which discards the possible imaginary part, as we are working with complex valued functions. For consistency and to ensure convergence, the acceleration parameter is forced to reside inside the interval $0< \alpha _k < 1$. At each iteration, we start with the current estimation of the phase $\phi _i$. Then, we compute the acceleration parameter and gradient to predict the closest possible phase to the next iteration, $y_k$. To this estimation, we apply our algorithm, $\psi (y_k)$, to obtain the next point phase estimation, $\phi _{k+1}$.

### Experimental implementation

**Figure 1:**
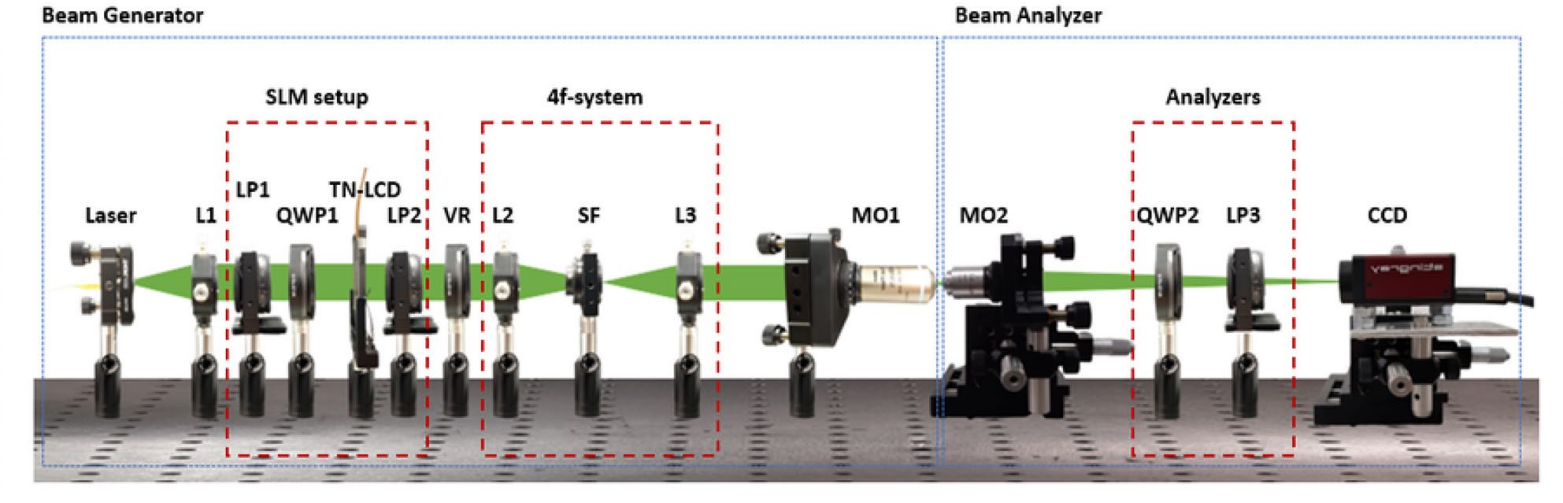
Experimental setup. L, LP, QWP, TN-LCD, SF, MO and CCD stand for lens, linear polarizer, quarter wave plate, Twisted nematic liquid crystal display, spatial filter, microscope objective and charge-coupled device, respectively.

The experimental system used in this work is divided in two parts. First, a beam generator able to produce highly focused fields with arbitrary irradiance and phase distribution (Fig. 1, left blue box). Second, a beam analyzer used to retrieve the transverse Stokes images of the produced beam (Fig. 1, right blue box).

A collimated beam is obtained after lens $\hbox {L}_1$ placed at a distance equal to focal length from the fiber end of a pig-tailed laser (Thorlabs LP520-SF15$\,@\,520\,\hbox{nm}$). The beam is modulated with a translucent twisted-nematic liquid crystal display (Holoeye HEO 0017), with a pixel pitch of $32 \upmu\hbox{m}$. Linear polarizers $\hbox {LP}_1$ and $\hbox {LP}_2$ and quarter wave plate $\hbox {QWP}_1$ are set to achieve a phase-mostly modulation response^64^ able to produce computer generated holograms according to the Arrizon’s Double-Pixel Hologram approach^65^. In order to produce radially polarized beams, we set a vortex retarder VR after polarizer $\hbox {LP}_2$. This element might be replaced with a QWP or simply removed to generate circularly or linearly polarized beams. Lens $\hbox {L}_2$ and $\hbox {L}_3$ form a telecentric (4f) system. A spatial filter is placed at the back focal plane of $L_2$. This element is required to remove high orders of the diffracted beam produced by the encoded hologram. An extended description on the use of the spatial filter in combination with Arrizon’s holograms can be found in^12,28^. The use and generation of holograms with this method in the context of high NA optical systems, as well as the intrinsic limitations due to the sampling of the modulation points, can be found in^34^. Then, microscope objective $\hbox {MO}_1$ (Nikon Plan Fluorite N40X-PF with NA$=0.75$) is used to focus the tailored beam and produce a highly focused beam.

The generated beam is imaged on a CCD camera (Stingray with a 14 bit depth and a pixel pitch of $3.75\,\upmu\hbox{m}$) by means of microscope objective $\hbox {MO}_2$ (Nikon with NA = 0.8) mounted on a movable stage, which is driven by a motorized device (Newport LTA-HL) with a uni-directional repeatability of $\pm 100\,\hbox{nm}$. Note that $\hbox {MO}_2$ should have a larger NA than the $\hbox {MO}_1$ in order to be able to collect all the beam. In this way, a set of observation planes separated by $2\,\upmu\hbox{m}$ are recorded. The magnification (M) and resolution of the beam analyzer part (Fig. 1, right blue box) is measured by imaging a 1951 USAF resolution test placed in front of $\hbox {MO}_2$, resulting in M=50*x* and a spatial sampling of $75\,\hbox{nm}$.

As described in “Longitudinal and transverse components of an electromagnetic field” and “Methods” sections, the longitudinal component of the electric field can be inferred from the two complex amplitudes of the transverse electric field, ${\mathbf {E}}_\perp = (E_x, E_y)$ [see Eq. (12)]. This means that the phase recovery algorithm should be independently used for the set of recorded irradiances $|E_x|^2$ and $|E_y|^2$ and thus, phases $\phi _x$ and $\phi _y$ can be obtained. Note that analyzers are placed in a zone where the beam can be considered under paraxial conditions. Therefore, $\hbox {LP}_3$ acts as a projector^66^ and can be used to select the proper direction of polarization of ${\mathbf {E}}_\perp$; the use of quarter wave plate $\hbox {QWP}_2$ is explained in the next paragraph.

Note that there is an arbitrary constant phase factor for each component that has no effect on the propagation of each amplitude alone, i.e. the relative phase delay between the two transverse components is arbitrary. However, since this relative phase is relevant for the longitudinal component estimation, we experimentally retrieve it by recording the corresponding Stokes images. A set of six polarimetric images is taken for each observation plane using quarter wave plate $\hbox {QWP}_2$ and linear polarizer $\hbox {LP}_3$ in front of the camera plane. Denoting by ${\beta , \theta }$ the phase delay introduced by the quarter wave plate and the rotation of the polarizer axis respectively, the Stokes measurements $I_{\alpha , \beta }$ are: $I_{0, 0}\ ;\ I_{0, 90}\ ;\ I_{0, 45}\ ;\ I_{0, 135}\ ;\ I_{\pi /2, 45}\ ;\ I_{\pi /2, 135}$. Finally, the relative phase delay $\delta$ is evaluated by

17 $$\begin{aligned} \tan \delta =\frac{S_3}{S_2}=\frac{I_{\pi /2, 135}-I_{\pi /2, 45}}{I_{0, 45}-I_{0, 135}}, \end{aligned}$$

whereas the amplitudes $|E_x|$ and $|E_y|$ are

18 $$\begin{aligned} |E_x|=\sqrt{I_{0, 0}}\; \mathrm { and }\; |E_y|=\sqrt{I_{0, 90}} \end{aligned}$$

## Results and discussion

Two different beams have been generated to estimate their corresponding longitudinal component. The first one is a radially polarized beam with a vortex phase :

19 $$\begin{aligned} \mathbf {E}_i(\mathbf {\rho }) = \left[ \cos \phi ,\ \sin \phi \right] ^T \rho \, \mathrm {e}^{i\phi }\mathrm {e}^{-\frac{\rho ^2}{f^2\mathrm {NA_e}^2}} \mathrm {circ}\left( {\frac{\rho }{f\mathrm {NA_e}}}\right) , \end{aligned}$$

where $\mathbf {\rho } = (x, y, 0)$, $\phi$ is the azimuth coordinate, and $f = 5$ mm is the focal length of MO1. $\mathrm {NA_e}$ is the effective pupil size of the beam which is determined according to the present size of the beam. The second one is a linearly polarized (1,1)-Hermite–Gauss beam:

20 $$\begin{aligned} \mathbf {E}_i(\mathbf {\rho }) = \left[ 0,\ \mathrm {H_1}\left( \frac{\sqrt{2}x}{f\mathrm {NA_e}}\right) \mathrm {H_1}\left( \frac{\sqrt{2}y}{f\mathrm {NA_e}}\right) \right] ^T\mathrm {e}^{-\frac{\rho ^2}{f^2\mathrm {NA_e}^2}} \mathrm {circ}\left( {\frac{\rho }{f\mathrm {NA_e}}}\right) \end{aligned}$$

**Figure 2:**
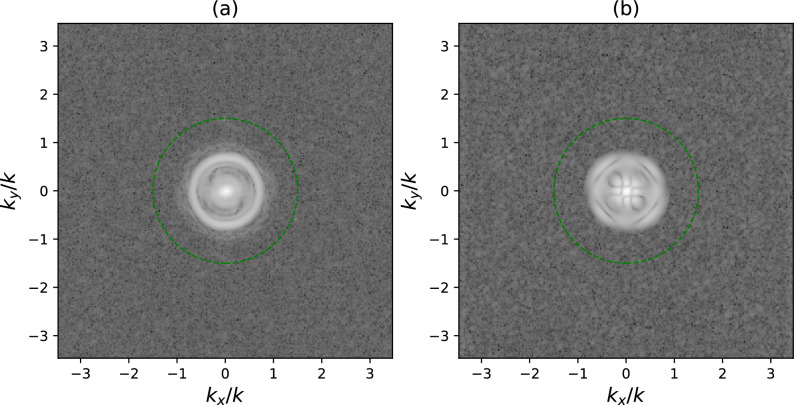
Modulus of the Fourier Transform of the intensity for **a** the radially polarized vortex beam and **b** the Hermite Gauss beam in logarithmic scale. The green circle indicates the theoretical maximum frequency allowed by the EP of the microscope objective.

The exact size of the beam at the EP of objective lens $\hbox {MO}_1$ is difficult to assess. Note that the aperture of each optical element limits the size of the beam as it propagates and the shape of the beam is modified by means of the computer generated hologram displayed on the liquid crystal display. Moreover, the EP of the objective is not physically accessible nor measurable. As the spectra of each component is limited by the physical size of the EP of the microscope, all generated beams are band limited. Then, as the Fourier Transform of the intensity can be demonstrated to contain up to twice the frequency content of the amplitude, we use its size to determine the effective pupil size that each beam uses. This calculation gives us $\hbox {NA}_e = 0.406$ and $\hbox {NA}_e = 0.379$ for the radially polarized vortex and the Hermite–Gauss beams respectively. We have NA$= 0.75$ for MO1, for reference, meaning that the beams do not completely fill the EP of the objective. The recorded transverse irradiance distributions of these beams after propagation through the optical system, using the electromagnetic propagation theory of Richards and Wolf^57^, is presented in Fig. 2.

**Figure 3:**
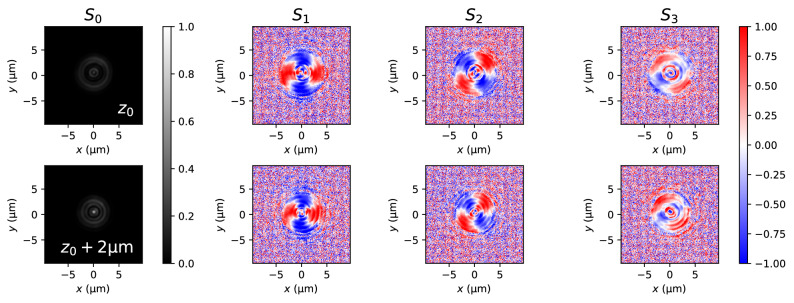
Stokes parameters for the radially polarized vortex beam measured at two planes perpendicular to the optical axis. The distance among these two planes is ${2}\,{\upmu \hbox {m}}$.

Figure 3 shows the Stokes parameters for two experimentally measured planes (first and second rows) for the radially polarized vortex beam case. The distance between these two planes is ${2}\,{\upmu \hbox {m}}$. Smaller distances proved to converge to a spherical wave independently on the shape of the beam. Note that the position $z_0$ is close to the focal plane, but cannot be easily set due to the difficulty to determine the focal plane with enough precision. We will numerically estimate its position as the plane where the energy of the beam is tightly concentrated. We observe that the polarization state of the beam is a complex combination of radial and circular polarizations distributed along the width of the beam. Moreover, we note that the exact polarization state of the beam changes as it propagates, specially near the center of the beam. This can be attributed to the spiral phase of the beam, which curls and uncurls while changing the phase difference between components.

**Figure 4:**
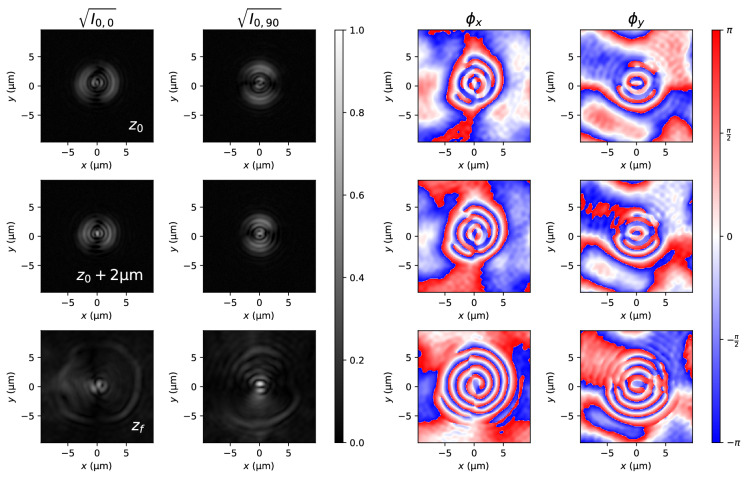
Vortex beam: Amplitudes and phases of the two experimentally observed planes (first two rows) and the synthetically refocused focal plane (bottom row).

Figure 4 (first and second rows) shows beam amplitudes $\sqrt{I_{0,0}}$ and $\sqrt{I_{0,90}}$ at planes $z_0$ and $z_0+2\,\upmu\hbox{m}$ . Using the phase retrieval algorithm (“Phase recovery algorithm” section), we obtained the corresponding phases $\phi _x$ and $\phi _y$. However, due to recovering each phase separately, the origin of phases for both $\phi _x$ and $\phi _y$ might be different. We have determined this constant random phase difference $\delta _0$ at the maximum of irradiance of the beam $\mathrm {max} \left( I_{0,0} + I_{0,90} \right)$ to be the one given by the Stokes parameters (Fig. 3), $\delta _0 = \arctan S_3/S_2$. With this information, the beam is propagated up to he focal plane. It can be observed that the focal plane is also the position where the phase has its simplest form, as a series of concentric ramification points. We must also remark that the phase difference in the focal plane is $\phi _y-\phi _x = 0.062$ rad, in contrast with its theoretical value, $\phi _y-\phi _y = \pi /2$ rad. This discrepancy may be due to some optical component introducing an uncontrolled phase difference between components. To compensate for this experimantally observed discrepancy, we include the phase difference between theory and experiment into the simulations of the beam in Eq. (19).

**Figure 5:**
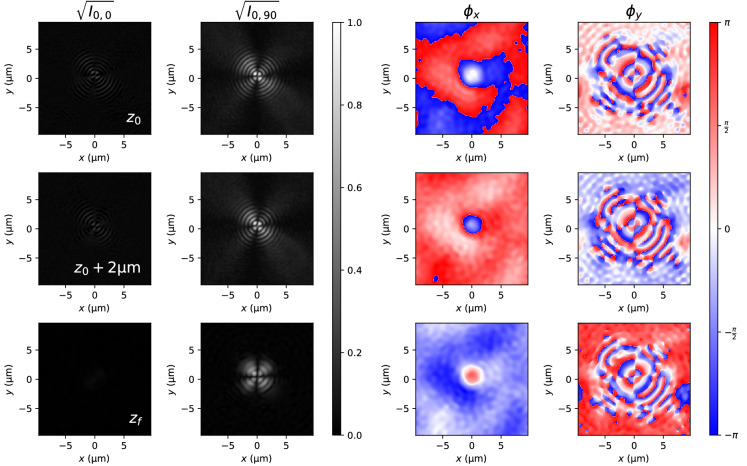
Hermite–Gauss beam: Amplitudes and phases of the two experimentally observed planes (first two rows) and the synthetically refocused focal plane (bottom row).

As the Hermite–Gaussian beam is linearly polarized, calculation of the Stokes parameters is unnecessary, and the amplitude is readily obtained from the irradiance. Figure 5 shows the *x* and *y* amplitudes of the experimentally measured beam and the synthetically refocused at the focal plane. The *x* component, although present, is very weak in comparison with the *y* component and does not affect the shape of the total irradiance. Its presence can be attributed to a failure of the analyzer to completely absorb the strong *y* component.

### Longitudinal component estimation

**Figure 6:**
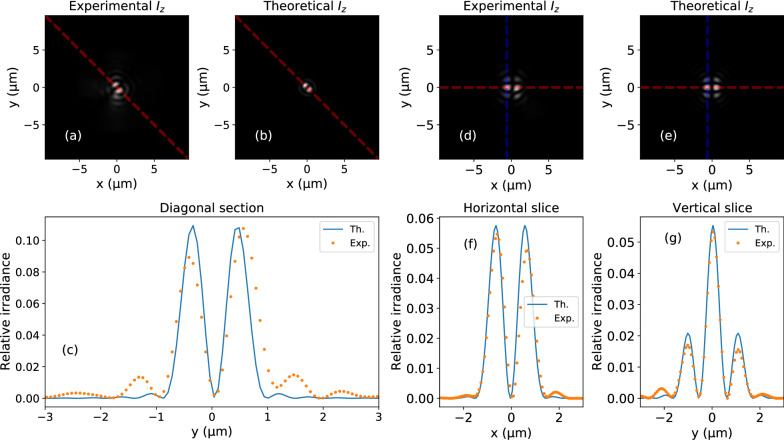
Estimation and theoretical irradiances at the focal plane of the microscope objective: (**a**–**c**) vortex beam: (**a**, **b**) 2D irradiance distribution; (**c**) profile of the irradiance across the diagonal depicted in red. The blue solid line and the orange dots corresponds to the theoretical and experimental values respectively. (**d**–**g**) Hermite–Gauss beam: (**d**, **e**) 2D irradiance distribution; (**f**, **g**) irradiance profiles across the horizontal and vertical lines superimposed on the experimental image. As in the previous case, orange points represent values obtained from experimental measures whereas the blue line has been obtained from theoretical calculations.

Provided that the complex amplitude of the transverse vector $\mathbf {E}_\perp (\mathbf {k}_\perp ; z=0)$ is known, Eq. (12) can be used to estimate the longitudinal component $E_z(\mathbf {k}_\perp ; z=0)$. Figure 6 summarizes the longitudinal component outcomes for the two beams considered. We observe a remarkable agreement between theoretical and experimentally estimated distributions: both show a similar size and shape.

As previously discussed, our beam does not strictly fulfill Eq. (19) due to an unforeseen phase difference introduced by some optical element in the experimental setup. This results in Fig. 6a, b, where two protruding lobes at 45 degrees can be observed. Figure 6c shows the intensity across the diagonal depicted in red, demonstrating a good agreement between experiment and theory. The experimental distribution is somewhat wider, has stronger secondary maxima and is slightly asymmetric with respect to the diagonal. These effects can be attributed to the imperfections in the hologram at the EP of the optical system, mainly due to the double pixel encoding technique used. Since the Hermite–Gauss beam is linearly polarized [Fig. 6d–g], there is no need to carry out the Stokes analysis as in the previous case. For this reason, it is noticeable an excellent agreement between theoretical and experimental results. Their size, principal maxima and secondary maxima coincide and are almost equal. Some dissimilarities can be observed in the irradiance in the xy plane, Fig. 6f, as the experimental beam is not perfectly symmetrical with respect to the $y = 0$ (red) line. The profiles shown in Fig. 6g are also very similar as well. As in the case of the vortex beam, discrepancies can be explained mainly due to the double pixel encoding used in the generation of the hologram. We do not have access to a continuous set of modulation points, but a limited number of them, as we discussed in^34,67^. Moreover, they are not uniformly distributed in the unit circle, which might cause a non-uniform error distribution across the modulation points^12,34^.

## Concluding remarks

In this work we have described a method to visualize the longitudinal component of a highly focused beam. The method is based on the characterization of the complex amplitude of the two transverse components of the focused beam in various planes: whereas the irradiance is recorded by means of a camera, the phase is estimated using phase recovery techniques. We realized it is also required to consider the relative phase between the two transverse components. This extra measurement can be carried out by determining the Stokes parameters of the beam. Finally, this information allows us to estimate the longitudinal component with the help of the Gauss theorem. The results obtained in the two cases analyzed show an excellent agreement between theory and experiments.
